# Occupational Laboratory Exposures to *Burkholderia pseudomallei* in the United States: A Review of Exposures and Serological Monitoring Data, 2008–2024

**DOI:** 10.3390/pathogens14090897

**Published:** 2025-09-05

**Authors:** Brian T. Richardson, Mindy G. Elrod, Katherine M. DeBord, Caroline A. Schrodt, Julie M. Thompson, Tina J. Benoit, Lindy Liu, Julia K. Petras, David Blaney, Jay E. Gee, Vit Kraushaar, Danielle Stanek, Katie M. Kurkjian, LaToya Griffin-Thomas, W. Gina Pang, Kristin Garafalo, Catherine M. Brown, Maria Bye, Christina Egan, Maria E. Negron, William A. Bower, Alex R. Hoffmaster, Zachary P. Weiner, Caitlin M. Cossaboom

**Affiliations:** 1 National Center for Emerging and Zoonotic Infectious Diseases, Centers for Disease Control and Prevention, Atlanta, GA 30329, USAnrm9@cdc.gov (C.M.C.); 2 Epidemic Intelligence Service, CDC, Atlanta, GA 30329, USA; 3 California Department of Public Health, Sacramento, CA 95814, USA; 4 Florida Department of Health, Tallahassee, FL 32311, USA; 5 Virginia Department of Health, Richmond, VA 23219, USA; 6 Career Epidemiology Field Officer Program, Division of State and Local Readiness, Office of Readiness and Response, Centers for Disease Control and Prevention, Atlanta, GA 30329, USA; 7 Pennsylvania Department of Health, Harrisburg, PA 17120, USA; 8 New Jersey Department of Health, Trenton, NJ 08625, USA; 9 Massachusetts Department of Public Health, Boston, MA 02130, USA; 10 Minnesota Department of Health, Saint Paul, MN 55155, USA; 11 New York State Department of Health, Albany, NY 12208, USA

**Keywords:** *Burkholderia pseudomallei*, indirect hemagglutination assay, serology, laboratory exposure, melioidosis, seroconversion

## Abstract

Infection with *Burkholderia pseudomallei*, the causative agent of melioidosis, is uncommon in the United States (U.S.), leading to delays in pathogen identification and clinical diagnosis which can often lead to laboratory exposures. The indirect hemagglutination assay (IHA) is the primary serological test for confirming exposure to *B. pseudomallei*. In the U.S., a titer of ≥1:40 suggests exposure to *B. pseudomallei* or a closely related species, and a 4-fold rise in IHA titer ≥1:40 with clinically compatible illness is considered diagnostically probable. A retrospective analysis of 160 voluntarily reported laboratory exposure events to *B. pseudomallei* across 29 U.S. jurisdictions and 5 countries between 2008–2024 was conducted. This analysis included post-exposure management data and IHA results for 855 exposed laboratory personnel who had serological monitoring performed at the U.S. Centers for Disease Control and Prevention (CDC). Among exposed laboratory personnel, 105 (12%) had a seropositive titer. Of these, ninety-one (87%) laboratory personnel remained seropositive (≥1:40) at their last IHA test. Five (1%) people had a 4-fold rise in titers, though none developed melioidosis. This report underscores the need for prospective studies to evaluate seropositive laboratory personnel and to update risk guidance for laboratory exposures in non-endemic areas.

## 1. Introduction

Melioidosis, an often-serious illness caused by the saprophytic bacterium *Burkholderia pseudomallei*, is primarily endemic to tropical and subtropical regions worldwide. This bacterium can infect humans and animals through direct skin contact with contaminated soil or water, as well as by inhalation, inoculation, or ingestion; however, person-to-person transmission is rare [1,2,3]. Clinical manifestations are wide-ranging, from localized infection, pneumonia, or bacteremia/disseminated infections, and often mimic other conditions [1]. Symptom onset generally occurs from 1 to 21 days post-exposure, but melioidosis may remain latent from months to years [1,4,5,6]. People with comorbid conditions, such as diabetes, hazardous alcohol use, and chronic liver disease, are at increased risk of symptomatic melioidosis [7,8]. Worldwide, an estimated 165,000 cases occur annually, predominately in hyperendemic Southeast Asia and northern Australia [9]. However, in the United States (U.S.), melioidosis is rare, with approximately 12 cases reported to the U.S. Centers for Disease Control and Prevention (CDC) annually, primarily travel associated [10,11]. Consequently, melioidosis is rarely considered in the differential diagnosis in the U.S., which may lead to inadvertent exposure of clinical diagnostic laboratory personnel to *B. pseudomallei* before it is identified [12,13].

Two published case reports detailed laboratory-acquired melioidosis following breaches in good laboratory practices [14,15,16]. Laboratory procedures, especially aerosol-generating activities performed without appropriate containment in a class 2 biosafety cabinet (BSC) or adherence to biosafety level 3 (BSL3) practices, pose the greatest risk. These procedures could cause inhalation, ingestion, and mucous membrane contact and have been implicated in cases of infection [16,17,18]. Compared to clinical diagnostic laboratories, research-based non-clinical laboratories are typically more aware of suspected specimens submitted for testing, which may result in fewer reports of accidental exposures to *B. pseudomallei* [19].

Since 2008, in response to laboratory exposures, CDC’s Bacterial Special Pathogens Branch (BSPB) provides technical assistance to jurisdictional partners. This includes assisting in identifying potentially exposed people, conducting risk assessments, and recommending post-exposure management, such as post-exposure prophylaxis (PEP) and serological monitoring. In 2009, CDC’s laboratory began conducting the indirect hemagglutination assay (IHA), a serologic test that may be useful in non-endemic regions for identifying exposed laboratory personnel, by detecting antibodies against *B. pseudomallei*. An increase in antibody titer suggests a recent exposure. In the U.S., a 4-fold rise in IHA titers in a symptomatic person is considered probable for melioidosis, though additional diagnostics like culture are necessary for confirmation [20,21].

*B. pseudomallei* is a Tier 1 overlap select agent, subject to strict reporting for exposures or releases outside of primary containment via the Animal and Plant Health Inspection Service (APHIS)/CDC Form 3 (https://www.selectagents.gov/forms/docs/APHIS-CDC_Form_3_English_Fillable.pdf (accessed on 18 August 2025)) to the CDC’s Division of Regulatory Compliance and Science (DRSC) [19,22]. Previous studies have contributed to our understanding of *B. pseudomallei* in the U.S.; for example, Benoit et al. [10] analyzed 47 suspected melioidosis cases and occupational exposures reported to BSPB from 2008 to 2013. More recently, DRSC reviewed 51 APHIS/CDC Form 3 release reports involving *B. pseudomallei* from 2017–2019. DRSC described exposure events, geographical locations, differences between registered entities (REs) and non-registered entities (NREs), and the root causes of release events [19,22]. While detailed, this report was limited to a 3-year timespan and both reports lacked data on serological testing.

Our analysis aims to comprehensively describe occupational laboratory exposures to *B. pseudomallei* in the U.S. from 2008 to 2024 that prompted clinical surveillance, post-exposure management, and serological monitoring. By extending the timeframe, expanding the data analyzed, and including serological monitoring data, this study addresses gaps in previous reports. The potential for severe illness and the lengthy treatment course associated with melioidosis make accidental *B. pseudomallei* exposure an important concern among laboratory personnel. Current risk assessment guidance for melioidosis, outlined by Peacock et al. [18], has not been updated in almost two decades, and reported data on risk assessment is limited. This analysis presents our current knowledge, which will inform and aid in revisions to risk assessment guidance for accidental laboratory exposures, thus enhancing our ability to understand outcomes and mitigate risks to laboratory workers.

## 2. Materials and Methods

### 2.1. Data Sources and Collection

Laboratory exposure events involving *B. pseudomallei* are voluntarily reported to BSPB via email, fax, or phone consultation/reporting. These exposures are often identified during the investigations of probable or confirmed melioidosis cases. Upon identification, BSPB consults with jurisdictional and laboratory partners to assess the potential for accidental exposures and provide post-exposure management recommendations.

BSPB used a secure Access database (Microsoft Corporation, Redmond, WA, USA), the “Melio Intake Database” (MID), from 2008 to 2014 to systematically capture voluntarily reported *B. pseudomallei* laboratory exposure event data. The MID was not used after 2014. The MID contained laboratory exposure event information including laboratory details, geographic location of laboratories, number of known laboratory exposures, number of exposed people, and exposure risk classifications. The database also captured individual level information, including demographics, preexisting conditions, exposure activities, treatment, and clinical signs and symptoms.

Since 2009, BSPB’s Zoonoses and Select Agent Laboratory (ZSAL) has used a secure Microsoft Access database, the “Indirect Hemagglutination Assay Log” (IHAL) to store IHA results of people exposed to *B. pseudomallei* in laboratory exposure events from laboratories that requested serological monitoring at CDC. The IHAL contains line-level laboratory testing information, including laboratory details, sample collection dates, and test results, as well as patient identifiers such as age, date of birth, and sex. Currently, ZSAL is the only laboratory in the U.S. performing IHA for *B. pseudomallei* serological monitoring. The MID and IHAL databases were developed independently. As a result, the data from these sources were not always directly comparable.

The IHAL contained exposure event data only from laboratories that requested serological monitoring at CDC. To aid cross-database deduplication, we applied a blocking technique to the MID, filtering to include only laboratories that requested serological monitoring at CDC. Events lacking serological request information were manually reviewed. For deduplication, we performed exact matching on common variables (lab names, states, event year, unique identifiers, and number of people exposed). Events with more than three matching variable values were flagged as potential duplicates. Flagged events were manually reviewed for verification.

### 2.2. Risk Categorization

People’s exposure risks are categorized as high-risk or low-risk depending on the activities that led to exposure and the nature of the incident based on guidance from Peacock et al. [18]. High-risk exposure incidents include inhalation, inoculation, or aerosol exposures to eyes or mucous membranes, while low-risk incidents might include minor spills in a BSC or opening agar plates outside of a cabinet [18]. An additional risk category of “unknown” was used in the MID for people identified as having some risk, but their risk classification or activities leading to the exposure were not shared with BSPB.

### 2.3. Serological Monitoring

Serological monitoring is recommended for all exposed persons regardless of risk categorization [18]. ZSAL performs *B. pseudomallei* serology using an IHA that incorporates antigens prepared from two prevalent strains originating from Northeast Thailand and Northern Australia using previously described methods [23]. IHA is performed on serum samples from exposed people collected on the day of suspected exposure (day 0 or initial testing) and weeks 1, 2, 4, and 6. Following previously established protocols, a single antibody titer ≥ 1:40 was considered positive. Further, an increase in titer levels between samples typically suggests a recent *B. pseudomallei* exposure, and a reproducible ≥ 4-fold rise in antibody titers between acute and convalescent serum collected at least two weeks apart is recognized as a probable *B. pseudomallei* infection [13,24]. Seroreversion was defined as the last IHA titer being < 1:40 after earlier positive titers.

### 2.4. Analyses

From the MID, we analyzed laboratory exposure events reported to BSPB from 2008–2014, including laboratory exposure activities, post-exposure management (treatment), number of exposed people, number of known laboratories involved, number of laboratory exposure events, geographic location of laboratories, and exposure risk classifications. Analyzed data between 2009–2024, from the IHAL, included age, sex, number of laboratory exposure events, geographic location of laboratories, exposure type, clinical status, and IHA titer results. Descriptive statistics, including counts, percentages, medians, and interquartile ranges (IQR) were calculated for all variables when appropriate. Associations between sex, age groups, and titer results were assessed using chi-square (χ^2^) tests and two-sided P values less than 0.05 were considered statistically significant. Analyses were conducted separately for total people tested and seropositive people. We completed analyses in SAS version 9.4 (SAS Institute Inc., Cary, NC, USA).

## 3. Results

### 3.1. MID Laboratory Exposure Events

**Figure 1:**
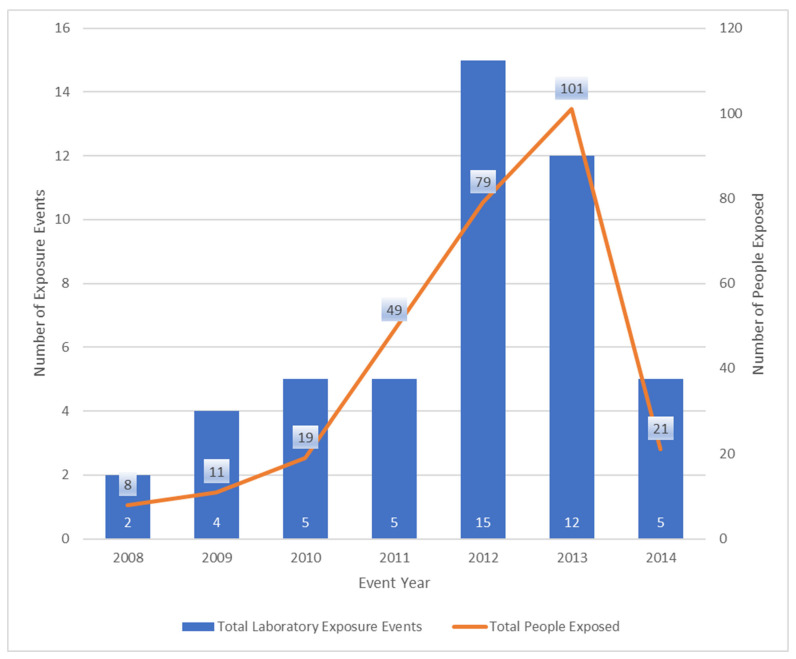
Number of *Burkholderia pseudomallei* laboratory exposure events and people exposed, by year: Melioidosis Intake Database (MID), United States, 2008–2014.

From 2008 to 2014, BSPB was notified of 81 instances of laboratories receiving a sample for identification that was later identified as *B. pseudomallei* across 19 jurisdictions. Of these, 48/81 (59%) resulted in a *B. pseudomallei* accidental laboratory exposure event, 26/81 (32%) resulted in no laboratory exposure event, and 7/81 (9%) did not provide additional information on if a potential exposure event occurred (Figure 1). The 48 laboratory exposure events occurred in 36 different laboratories, and four laboratories accounted for 32% (16/48) of all exposure events. When comparing the number of people exposed per event, 14 of 48 (29%) events involved one exposed person, 29 (60%) events involved between 2 to 10 exposed people, and 5 (10%) events involved more than 10 exposed people. As shown in Figure 1, the number of exposure events fluctuated, with the highest number of events reported in 2012 (n = 15), involving 79 people, and the highest number of people exposed (n = 101) occurring in 2013 from 12 events.

Across the 48 laboratory exposure events, 288 people were identified as having a laboratory exposure to *B. pseudomallei*, of which 16% (n = 46) were classified as having a high-risk activity, 53% (n = 153) as low-risk, and 31% (n=89) as unknown risk. Among low-risk activities, inadvertent opening of the lid of an agar plate growing *B. pseudomallei* outside a BSC was the most reported activity (Table 1). The risk of generating aerosols outside of a BSC was associated with nearly all high-risk exposures. Most activities that led to exposure were performed outside of a BSC, which accounted for 69% (n = 38/55) of all reported exposure activities. All high-risk activities (n = 20/20, 100%) were performed outside of a BSC and 46% (n = 18/35) of low-risk activities were performed outside of BSC.

PEP was offered in half (n = 24/48, 50%) of the exposure events; this included all high-risk and low-risk exposed persons with risk factors for melioidosis. Among exposed people, 22% (n = 66/288) opted to initiate PEP, 38% (n = 111/288) did not initiate PEP, and the PEP status of 38% (n = 111/288) was not reported to BSPB. Follow-up information on PEP regimen, completion rates, effectiveness, and outcomes of exposed people were not provided to BSPB. CDC offered to perform serological monitoring for everyone exposed in events reported to BSPB, of which, serum was sent to CDC’s ZSAL for 20 out of 48 (42%) events. Since IHA results were not captured in the MID, we reviewed data from the IHAL, and in the same timeframe, 2008–2014, ZSAL performed IHA on 830 serum samples from 347 exposed laboratory personnel, and 33 (10%) people were seropositive. IHA titers in those who were seropositive ranged from 1:40 to 1:160. There were two people who had a 4-fold rise ≥1:40; repeat titers for one person were stable <1:10 and stable at 1:40 for the other (Table 2). There were no probable or confirmed laboratory-acquired infections resulting from laboratory exposure events from 2008–2014. Overall, after deduplication and manual verification, a total of 10 laboratory exposure events and 74 people were confirmed duplicates across the MID and IHAL.

### 3.2. IHAL Laboratory Exposure Events and Serological Monitoring

From the IHAL database, between August 2009 and December 2024, CDC performed 2404 IHA tests on samples from 855 people exposed to *B. pseudomallei* in 122 laboratory exposure events from 29 U.S. jurisdictions and 5 international countries, including Canada, Belgium, Isreal, Norway and Switzerland. Most exposure events in the IHAL (n = 83/122, 67%) resulted in 2 to 10 exposed people undergoing seromonitoring at ZSAL; 21 (17%) events resulted in only one exposed individual and 20 (16%) events resulted in more than 10 exposed people. Of all IHA tests performed, 44% (n = 1069/2404) were from the 20 largest exposure events (>10 exposed people).

**Figure 2:**
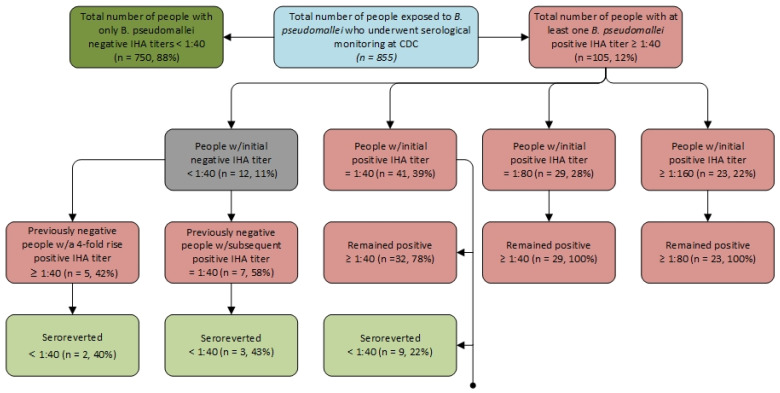
Flowchart of serological results for 855 people exposed to *Burkholderia pseudomallei* in U.S. laboratory exposure events: Indirect Hemagglutination Assay Log (IHAL), United States, 2009–2024.

In total, 105/855 (12%) people were seropositive (Figure 2). Among these people, IHA titers ranged from 1:40 to 1:320. Of the 105 seropositive people, 91/105 (87%) remained positive with repeat titers, while 14/105 (13%) seroreverted. People with an initial titer of 1:40 remained positive 78% of the time, whereas those with initial titers of 1:80 or 1:160 remained positive 100% of the time. All people who initially had negative titers but later tested positive subsequently seroreverted.

Most people (671/855, 78%) had between 2 and 5 serum samples tested. Among seropositive persons for whom sex was reported, 83 of 95 (87%) were female, accounting for 230 of 285 (80%) positive titers. Of all samples tested, 88% (n = 2119/2404) were seronegative and 12% (n = 285/2404) were positive. Fifty-seven percent (n = 162/285) of positive titers were ≥1:80. More IHA tests were performed for females (n = 1731/2404, 72%) than males (465/2404, 19%) (Table 3). A higher percentage of females (83/583, 14%) had at least one positive titer, compared to males (11/160, 7%), among those for whom sex was reported.

People aged 50–59 years accounted for the largest proportion (192/855, 22%) of exposed people who underwent seromonitoring, but the 30–39 year-old age group had the highest percentage of seropositivity (25%) among all exposed people. Of those who underwent symptom monitoring, all (356, 100%) were asymptomatic. We did not receive clinical status information for 499/855 (58%) exposed people.

### 3.3. IHAL Presumptive Laboratory Evidence: IHA 4-Fold or Greater Rise

There were 5/855 (<1%) people who had a 4-fold rise ≥1:40 in titers (Table 2). Two people seroreverted and three had elevated titers that remained stable, i.e., within one dilution of the highest titer but ≥1:40. Two people experienced a high-risk exposure from the same event while performing activities outside of a BSC. One person had a predisposing lung condition. Initially, both people declined PEP but received it later. The person with the predisposing lung condition seroreverted after six months, while the other person remained persistently positive over a year later.

## 4. Discussion

This report describes more than 2400 IHA tests from 850 exposed laboratory personnel from at least 47 laboratories. Spanning 16 years, it includes 160 occupational laboratory exposure events to *B. pseudomallei* across 29 U.S. jurisdictions and five countries. This report is the first to describe *B. pseudomallei* seropositivity among laboratory personnel with occupational exposures in the U.S. and internationally, with all IHA tests performed at CDC.

The IHA is the preferred serologic assay globally, despite a lack of standardization and semiquantitative results [3]. In melioidosis endemic regions, high background seropositivity reduces the diagnostic value and specificity of IHA and other serologic tests [20,25,26,27,28,29]. In Thailand, a study evaluating 188 healthy blood donors showed IHA titers of ≥1:40, with specificity for this cutoff at 67.6% [30]. Higher cutoff values (e.g., 1:160) have been used in endemic areas to improve specificity [25]. In contrast, studies show specificity improves substantially in non-endemic populations, reaching 98–100% for serological methods collectively [27,30,31]. When evaluated with serum from 90 healthy U.S. donors, the IHA demonstrated 100% specificity, reinforcing that in non-endemic regions, positive serology, as observed in Figure 2, is more likely to reflect true exposure [30]. Thus, the IHA is particularly useful for assessing laboratory personnel and travelers for exposure, though culture confirmation remains necessary for diagnosis [18,29].

IHA titer seropositivity cutoff values may vary widely (1:10 to 1:160) by country or laboratory [25,32]. Gassiep et al. [28] considered titers ≥1:10 as the threshold for potential exposure, with titers 1:10 and 1:20 classified as borderline, and ≥1:40 as positive for culture confirmed cases. While IHA titers ≥1:40 in our report may suggest exposure, false-positives can occur due to non-specific reactions. Although repeat IHA testing on additional samples is recommended, testing was not always feasible for BSPB [18]. Nonetheless, all IHA testing in this report was performed in one lab using the same assay and cutoff values as described. The consistency in testing methodology and interpretation strengthens the reliability of our serological findings.

A large proportion of laboratory exposure activities, 65% of all activities (100% of high-risk and 46% of low-risk activities), were performed outside of a class II BSC. Clay et al. [19] reported that up to 84% (43/51) of release events in U.S. laboratories were performed outside of BSCs. Despite no melioidosis cases being reported by Clay et al. [19] or in our report, it is noteworthy that most exposures in both reports occurred outside of primary containment. Notably, two instances of laboratory-acquired melioidosis were associated with activities conducted outside of a BSC or primary containment, presumably by aerosol inhalation [14,15]. BSCs are the most effective tool for prevention of laboratory-acquired infections [33].

Most reported exposure events involved multiple people. Contributing factors included performing activities outside of a BSC, especially in clinical laboratories where *B. pseudomallei* may not be initially suspected. Additionally, the close working proximity (within 5 feet) of colleagues to others manipulating enriched material, combined with a lack of clinical suspicion during sample submission, can facilitate these exposures. Given these risks, laboratory personnel should be well-aware of potential hazards during routine activities outside of a class II BSC and receive institutional biosafety training that explicitly outlines necessary precautions.

Currently, there is a paucity of published data on *B. pseudomallei* seropositivity rates among people exposed during routine laboratory procedures. In this report, 12% of people were seropositive following laboratory exposure to *B. pseudomallei*, with 47% showing at least one detectable titer (≥1:10). Our analysis identified a relatively high seroprevalence rate, even without concurrent clinical symptoms. This rate exceeded those reported outside of laboratory settings in the ultra-endemic region of Northern Australia (5.6%), and among people in northern Queensland, Australia, with known melioidosis risk factors such as diabetes (8.5%) [20,34]. A positive IHA titer may indicate true exposure, but false positives can occur from cross-reactivity with antigenically similar organisms such as *Burkholderia thailandensis* or other *Burkholderia* spp., non-specific reactions, or non-occupational exposures. While IHA seropositivity (≥1:40) in non-endemic regions is more likely to reflect genuine exposure due to higher specificity, results should be interpreted carefully, particularly without clinical symptoms or culture confirmation [27,30,31,35]. Notably, 87% of seropositive laboratory personnel with repeat titers remained positive; however, all were asymptomatic, and no laboratory personnel developed melioidosis. No confirmed melioidosis cases were reported in association with a documented laboratory exposure.

In this report, the proportion of seropositive females was twice that of males (14% vs. 7%), with females accounting for 78% of all positive tests. The highest percentages of seropositivity were among the 30–39 year-old (25%) and 20–29 year-old (24%) age groups (Table 3); however, there was no statistically significant association between age group and seropositivity. This is consistent with studies from Eastern India, where seropositivity was highest among people aged 21–30 years-old [36]. A Thai study found people aged ≥45 years were less likely to be seropositive than those <45 years [25,26]. While some studies observed age-specific increases up to the age of 20, others found no significant association between age and seropositivity [37,38,39]. Higher seropositivity among younger age-groups across locations and exposure sources might suggest an underlying biological susceptibility that varies with age. However, confounding factors such as occupational patterns, frequency or level of exposure, and behavior patterns may influence these differences.

The IHAL only included laboratory personnel who voluntarily underwent serological monitoring, potentially introducing participation bias. People with higher perceived risk or experiencing symptoms may have been more likely to participate, skewing results and potentially overestimating seropositivity in some populations. Although testing distribution by sex (69% female, 19% male) was similar to national estimates for clinical laboratory professionals (75% female, 25% male) [40], the observed age and gender differences in testing and seropositivity may reflect workforce demographics, serological monitoring participation, occupational patterns, or unmeasured confounders. Furthermore, the findings are descriptive only of included laboratory personnel and are not generalizable to the broader laboratory workforce or other healthcare settings.

Of the 105 seropositive people, 87% (91/105) had positive initial acute titers. Among all positive titers, 57% were ≥1:80 and 26% were ≥1:160. Repeat titers remained positive in 81% of people with initial positive titers. Persistent low and high level IHA seropositivity have been described in the literature, but implications for disease acquisition and outcomes remain unclear. Five people had a titer result showing a 4-fold rise ≥1:40. In the U.S., a clinically compatible illness with a 4-fold rise ≥1:40 would be considered a probable case, whereas in Thailand a single IHA titer ≥1:160 with clinically compatible illness is regarded as probable [21,41]. Nonetheless, even among the five people who had a 4-fold rise in IHA titers, no one in this report was found to have an illness clinically compatible with melioidosis.

This report has limitations. A primary limitation being that reporting accidental laboratory exposures to *B. pseudomallei* to BSPB is not mandatory. This is evidenced by the discordant number of events reported between the IHAL (25) and DRSC (51) from 2017–2019 [19]. For example, clinical surveillance was offered to 275 people per DRSC, while 242 people underwent serological monitoring at CDC per the IHAL. Consequently, voluntary reporting limited the completeness of event reporting and information sharing, which often only occurred when technical assistance or serological monitoring was requested.

Both IHAL and MID have specific limitations. The MID captured event-level information only when technical assistance was requested, and lacked individual post-exposure management data. Similarly, the IHAL included data only from those undergoing serological monitoring at CDC, and did not capture details regarding exposure activities, risk classifications, or post-exposure management. These limitations, combined with a lack of standardization across databases, hindered direct analysis and comparison of most exposure activities and post-exposure management (e.g., symptom monitoring, serological monitoring, PEP). Prospective studies should aim to collect comprehensive data in a single, standardized database to improve surveillance and allow deeper analysis. Furthermore, the seroconversion date could not be determined because exposure dates and baseline (pre-exposure) serological samples were rarely provided to BSPB. Additionally, clinical monitoring information was not reported for 58% of exposed people, limiting our ability to accurately assess the incidence of symptomatic melioidosis and the relationship between seropositivity and clinical outcomes. This may have led to underestimation of disease incidence among exposed.

These databases also lacked data on potential non-occupational exposures and comorbid medical conditions. Specific risk factors for *B. pseudomallei* exposure, such as travel to endemic regions, severe weather, country of origin, or certain /hobbies (e.g., gardening), were not captured. Exposure locations were excluded from reporting to maintain privacy of individuals and laboratories involved. Comorbid medical conditions were not captured. The absence of these data is significant, as these risk factors impact the likelihood of developing melioidosis after *B. pseudomallei* exposure and could explain the low melioidosis incidence among laboratory personnel observed in this report.

## 5. Conclusions

The outcomes and risk associated with melioidosis makes *B. pseudomallei* an important concern among laboratory personnel. While the seropositivity rate among exposed laboratory personnel was 12%, and most titers were comparatively high, it remains rare for laboratory personnel exposed to *B. pseudomallei* during laboratory testing to develop melioidosis. The seroconversion rates presented here may suggest self-limiting subclinical infections in laboratory personnel, which is common among most healthy people exposed to *B. pseudomallei*, although latent infections may also occur [1]. Laboratory exposures to *B. pseudomallei* may become increasingly frequent and cause public health challenges in the U.S. as *B. pseudomallei* was recently found to be endemic in the Gulf Coast of the U.S. in 2022 [42]. There are practical exposure prevention practices, which protect against exposure to *B. pseudomallei* and other pathogens during routine laboratory procedures. It is recommended that manipulation of any unidentified specimens occur in a BSC until infectious pathogens, especially select agents, are ruled out. Additionally, specimens from patients suspected of *B. pseudomallei* infection should be flagged for laboratories. Prospective longitudinal studies are needed to identify appropriate serological and symptom monitoring timelines, to evaluate clinical courses of seropositive exposed laboratory personnel, and to update risk assessment guidance for laboratory exposures to *B. pseudomallei* in non-endemic countries. Interpretation of this report should consider limitations of incomplete data and voluntary reporting. Ongoing surveillance, multidisciplinary research, and collaboration are important to protect laboratory personnel and address emerging risks associated with *B. pseudomallei.*

## Figures and Tables

**Table 1:** Laboratory activities potentially leading to exposure to *Burkholderia pseudomallei*: Melioidosis Intake Database (MID), United States, 2008–2014.

Activity	Potential Exposure Activity N = 55 (%)
**Low-Risk Activities**	
Inadvertent opening of the lid of an agar plate growing *B. pseudomallei* outside a biologic safety cabinet	18 (33)
Inadvertent sniffing of agar plate growing *B. pseudomallei* in the absence of contact between worker and bacterium	5 (9)
Splash event leading to visible contact of *B. pseudomallei* with gloved hand or protected body, in the absence of any evidence of aerosol	2 (4)
Spillage of small volume of liquid culture (<1mL) within a functioning biologic safety cabinet	1 (2)
Activity unspecified/unknown (to BSPB), but classified and reported to BSPB as low-risk	9 (16)
**High-Risk Activities**	
Splash event leading to contamination of mouth or eyes (outside of BSC)	1 (2)
Generation of aerosol outside biologic safety cabinet (e.g., sonication, centrifuge incident)	19 (34)

**Table 2:** Laboratory personnel (n = 5) with a 4-fold rise in IHA titer to ≥1:40: Indirect Hemagglutination Assay Log (IHAL), United States, 2009–2024.

Patient	Titer	Date Collected	Seroreversion	Exposure	Other Details
1	<1:10	05/08/2009	Yes		Low-risk exposure
	1:80	05/22/2009		Outside BSC	
	<1:10	06/19/2009			
2	1:10	03/04/2011	No	Outside BSC	Absence of risk factors, started on PEP
	1:10	03/11/2011			
	1:40	Unknown			
	1:40	Unknown			
3	1:10	12/19/2014	No	Unknown	Event led to 7 high-risk and 12 low-risk exposures
	1:10	12/26/2014			
	1:10	01/05/2015			
	1:10	01/16/2015			
	1:10	01/30/2015			
	1:40	06/21/2018			
	1:40	07/02/2018			
	1:20	07/16/2018			
	1:20	07/30/2018			
	1:40	12/04/2018			
4	<1:10	01/17/2019	Yes	Outside BSC	High-risk exposure, predisposing lung condition, declined PEP initially
	<1:10	01/23/2019			
	<1:10	01/30/2019			
	1:10	02/13/2019			
	1:160	02/27/2019			
	1:40	06/27/2019			
	1:10	08/21/2019			
5	<1:10	01/16/2019	No	Outside BSC	High-risk exposure, declined PEP initially
	1:40	01/23/2019			
	1:40	01/30/2019			
	1:40	02/13/2019			
	1:40	03/01/2019			
	1:20	06/07/2019			
	1:40	08/06/2019			
	1:40	09/16/2019			
	1:40	02/19/2020			

**Table 3:** Summary of indirect hemagglutination assay (IHA) tests (n = 2404) and laboratory personnel (n = 855) by, sex and age groups: Indirect Hemagglutination Assay Log (IHAL), United States, 2009–2024.

	IHA(-) Tests ^a^ <1:40	IHA(+) Tests ^a^ ≥1:40	Total IHA Tests	IHA(-) People ^b^ <1:40	IHA(+) People ^b^ ≥1:40	Total People	χ^2^ *p* Value ^c^
**Overall**	**n = 2119** **n (%)**	**n = 285** **n (%)**	**N = 2404** **n (%)**	**n = 750** **n (%)**	**n = 105** **n (%)**	**N = 855** **n (%)**	
**Sex**							
**Female**	1501 (71)	230 (80)	1731 (72)	500 (67)	83 (80)	583 (69)	0.0068
**Male**	437 (21)	29 (10)	465 (19)	149 (20)	11 (10)	160 (19)	
**Missing**	181 (8)	27 (9)	208 (9)	101 (13)	11 (10)	112 (12)	
**Age Group (yrs)**							
**Median (IQR^d^)**	43 (23)	40 (22)	43 (23)	43 (23)	40 (19)	42 (23)	
**20–29**	380 (18)	71 (25)	451 (19)	124 (17)	25 (24)	149 (17)	0.0718
**30–39**	437 (21)	62 (22)	499 (21)	152 (20)	26 (25)	178 (21)	
**40–49**	350 (17)	62 (22)	412 (17)	117 (16)	23 (22)	140 (16)	
**50–59**	526 (25)	56 (20)	582 (24)	176 (23)	16 (15)	192 (22)	
**60–69**	284 (13)	18 (6)	302 (13)	93 (12)	8 (8)	101 (12)	
**70+**	5 (<1)	0	5 (<1)	2 (<1)	0	2 (<1)	
**Missing**	137 (6)	16 (6)	153 (6)	86 (11)	7 (6)	93 (11)	
**Age Group (yrs)**							
**<45**	1005 (48)	158 (56)	1164 (48)	338 (45)	38 (36)	376 (44)	0.0527
**≥45**	977 (46)	111 (39)	1087 (45)	326 (43)	60 (57)	386 (45)	
**Missing**	137 (6)	16 (0)	153 (<1)	86 (12)	7 (7)	93 (11)	

^a^ IHA = Indirect Hemagglutination Assay; IHA(-) indicates the number of samples with negative titers (<1:40); IHA(+) indicates the number of samples positive titers (≥1:40). ^b^ IHA(-) indicates the number of people with negative titers (<1:40); IHA(+) indicates the number of people with positive titers (≥1:40). ^c^ P values are from chi-square (χ^2^) tests assessing associations between sex or age group and IHA status (IHA-negative vs. IHA-positive) at the person level. ^d^ IQR = Interquartile range.

## Data Availability

The datasets analyzed in this manuscript are historical data from CDC’s Bacterial Special Pathogens Branch and are not publicly available due to privacy and ethical restrictions. De-identified data may be available from the corresponding author upon reasonable request and with permission from the CDC. For further information, please contact the corresponding author.
